# Spotted fever group *Rickettsia*, *Anaplasma* and *Coxiella*-like endosymbiont in *Haemaphysalis* ticks from mammals in Thailand

**DOI:** 10.1007/s11259-022-09980-x

**Published:** 2022-08-09

**Authors:** Supanee Hirunkanokpun, Arunee Ahantarig, Visut Baimai, Pairot Pramual, Pakavadee Rakthong, Wachareeporn Trinachartvanit

**Affiliations:** 1 Department of Biology, Faculty of Science, Ramkhamhaeng University, Bangkok, 10240 Thailand; 2 Biodiversity Research Cluster, Department of Biology, Faculty of Science, Mahidol University, Bangkok, 10400 Thailand; 3 Department of Biology, Faculty of Science, Mahasarakham University, Maha Sarakham, 44150 Thailand; 4 Faculty of Science and Technology, Rajabhat Suratthani University, Surat Thani, 84100 Thailand

**Keywords:** Tick, SFG *Rickettsia*, *Coxiella*, *Anaplasma*, Burmese ferret-badger, Asian palm civet

## Abstract

Ticks are ectoparasites of vertebrates and vectors of various pathogenic microorganisms. In this study, the presence of bacteria and protozoa was evaluated by PCR and DNA sequencing in 233 mammal ticks collected from 8 provinces in Thailand. Sequence and phylogenetic analyses of partial rickettsial *ompA*, *ompB*, *sca4* and partial *Coxiella* 16S rRNA, *GroEL*, *rpoB* genes clearly revealed, for the first time, a co-infection of SFG *Rickettsia* belonging to *R. massiliae* subgroup and *Coxiella*-like endosymbiont (CLE), Cox-hein, in a male of *Haemaphysalis heinrichi* tick infesting Burmese ferret-badger in Loei province. Moreover, a male of *H. hystricis* tick infesting the same host was infected with another CLE, Cox-hys. Based on the 16S rRNA gene sequence, *Anaplasma* sp., closely related to *Anaplasma bovis* was also detected in a male of *H. heinrichi* infesting the same Burmese ferret-badger. In addition, the third CLE, Cox-asia, found in *H. asiatica* collected from Asian palm civet in Chiang Rai province, was different from both Cox-hein and Cox-hys. This study provided important data and broadened our knowledge on tick-borne pathogens and endosymbionts in Thailand and Southeast Asia.

## Introduction

Ticks are important vectors of infectious pathogens including bacteria, protozoa, and viruses. The obligate intracellular bacteria, *Rickettsia*, is notorious for causing infection and mild to severe diseases in humans and other mammals (Raoult and Roux 1997). Based on phylogenomic analyses, members of the genus *Rickettsia* are classified into four groups, namely, spotted fever group (SFG), typhus group (TG), transitional group (TRG), and ancestral group (AG) (Gillespie et al. 2007).

*Coxiella burnetii* is the etiological agent of a worldwide zoonotic disease called Q fever. In Thailand, infective endocarditis caused by *C. burnetii* has been reported (Pachirat et al. 2012). Both animals and humans can be infected by this bacterium, usually through inhalation of contaminated aerosols. Its reservoir hosts comprise mammals, birds, and arthropods, particularly ticks (Raoult and Marrie 1995). However, the role of ticks as the vector of *C. burnetii* remains controversial. Many Ixodidae and Argasidae ticks, including *Amblyomma*, *Dermacentor*, *Haemaphysalis*, *Ixodes*, *Rhipicephalus*, *Argas*, and *Ornithodoros* also harbor *Coxiella*-like endosymbionts (CLE) (Duron et al. 2015). Thus far, there is no evidence of the transmission of CLE by ticks to vertebrates. In addition to CLE, another endosymbiont commonly found in many arthropods and filarial nematodes is *Wolbachia* bacteria. In arthropods, *Wolbachia* has been reported to manipulate host reproduction including cytoplasmic incompatibility, parthenogenesis, feminization of genetic males, and male killing. Therefore, current research focuses on *Wolbachia* application to protect humans from vector-borne diseases (Werren et al. 2008).

*Anaplasma* and *Ehrlichia* are tick-transmitted bacteria in the family Anaplasmataceae, order Rickettsiales. *Anaplasma* is an obligate intracellular bacterium living in mammal blood cells. Members of this genus include tick-borne pathogens causing fatal infectious diseases in humans and catastrophic diseases in animals (Dumler et al. 2001). Anaplasmosis in humans is principally caused by *Anaplasma phagocytophilum*. *Anaplasma* has also been found in ticks of the family Ixodidae, including the genera *Amblyomma, Dermacentor, Ixodes,* and *Rhipicephalus* (Dumler et al. 2001). *Ehrlichia* spp. are obligatory intracellular bacteria that cause ehrlichiosis in animals and humans. In Thailand, several *Ehrlichia* spp. that are the etiologic agents of ehrlichiosis including *E. canis*, *E. chaffeensis*, and *E. platys*, have been reported (Parola et al. 2003; Pinyoowong et al. 2008).

Lyme disease is a tick-borne disease caused by *Borrelia burgdorferi*, whose main vectors are *Ixodes* ticks, i.e., *Ixodes ricinus* and *Ixodes scapularis*. This bacterial pathogen has also been reported to be carried by other tick genera, namely *Amblyomma*, *Dermacentor*, *Haemaphysalis*, *Hyalomma*, and *Rhipicephalus*. Moreover, other members of the genus *Borrelia* are known to be the cause of human diseases, for example, *B. afzelii* and *B. garinii* (Margos et al. 2009).

*Babesia* spp. and *Hepatozoon canis* are tick-transmitted apicomplexan parasites that are causative agents found in dogs, cattle, and wild animal species (Baneth 2011; Gray et al. 2019). Babesiosis is caused by several *Babesia* spp. which are transmitted by hard ticks such as *Rhipicephalus sanguineus*, *H. longicornis*, and *H.* *elliptica* (Gray et al. 2019). *Hepatozoon canis* is the causative agent of hepatozoonosis widely distributed in many countries (Baneth 2011). Although the brown dog tick, *R. sanguineus*, is the main vector of *H. canis*, this pathogen also infects several other tick species, e.g., *Ambylomma ovale*, *H. flava*, *H. longicornis*, *R. microplus*, and *R. turanicus* (Baneth 2011; Demoner et al. 2013; Giannelli et al. 2017).

The study of pathogens that are transmitted by ticks will be useful for understanding the role of ticks as vectors of human and other animal disease agents. This information is crucial for effective monitoring and control of tick-borne diseases. Thus, the aim of this study was to investigate the presence of bacteria and protozoa in mammal ticks collected from several regions in Thailand using molecular approaches.

## Materials and methods

### Tick collection and identification

A total of 233 ticks were collected from domestic and road-killed mammals (23 hosts belonging to 7 species) in 8 provinces of Thailand during 2015–2019 (Table 2). All tick samples were kept in labeled collection vials individualized per host, containing 70% ethanol. They were identified morphologically by using a stereomicroscope following previously published taxonomic keys (Cooley 1946; Wassef and Hoogstraal 1984; Tanskul and Inlao 1989). Species of ticks were molecularly identified by partial sequencing of 16S rRNA gene (Black and Piesman 1994) to confirm their morphological identifications.

### DNA extraction and PCR amplification

Each individual tick was rinsed in 10% sodium hypochlorite, 70% ethanol, and sterile distilled water three times (1 min each). Genomic DNA was extracted from individual adults, nymphs, pooled nymphs, and pooled larvae using the QIAamp DNA Extraction Kit for Tissue (QIAGEN) according to the manufacturer’s protocol. The presence of bacteria and protozoa, including *Anaplasma*, *Borrelia*, *Coxiella*, *Ehrlichia*, *Rickettsia*, *Wolbachia*, *Babesia*, and *Hepatozoon* in tick samples was initially screened by Polymerase Chain Reaction (PCR) using primers as shown in Table 1. For *Rickettsia* characterization, the primers Rr17.61p/Rr17.492n were initially used to amplify a 434 bp fragment of the 17-kDa antigen gene. Subsequently, the 17-kDa-positive samples were amplified and sequenced with primers specific to *gltA*, *ompA*, *ompB*, and *sca4* genes (Webb et al. 1990; Regnery et al. 1991; Roux and Raoult 2000; Jiang et al. 2005). For *Coxiella,* the 16S rRNA positive samples were further amplified with primers targeting *rpoB* (DNA directed RNA polymerase beta) and *GroEL* (60 kDa chaperone heat shock protein B) genes (Duron et al. 2014, 2015). Additionally, the positive sample tested with EHR16SD/EHR16SR primers was amplified and sequenced with *Anaplasma* 16S rRNA primers as previously described by Zobba et al. (2014) (Table 1).

**Table 1:** Primers used for PCR detection of bacterial/protozoal microorganisms and tick DNA

Target organism	Target gene	Primer name	Primer sequences (5´-3´)	Amplification fragment size (bp)	References
*Rickettsia* spp.	17-kDa antigen	Rr17.61p	GCTCTTGCAACTTCTATGTT	434	Webb et al. 1990
		Rr17.492n	CATTGTTCGTCAGGTTGGCG		
	Citrate synthase (*gltA*)	RpCS.877p	GGGGGCCTGCTCACGGCGG	381	Regnery et al. 1991
		RpCS.1258n	ATTGCAAAAAGTACAGTGAACA		
	190-kDa protein antigen (*ompA*)	Rr190.70p	ATGGCGAATATTTCTCCAAAA	532	Regnery et al. 1991
		Rr190.602n	AGTGCAGCATTCGCTCCCCCT		
	120-kDa protein antigen (*ompB*)	RicF	CAGCAAGGTAATAAGTTTAATAC	~ 800	Hirunkanokpun et al. 2022
		RicR	GCTATACCGCCTGTAGTAACAG		
	120-kDa cytoplasmic protein (*sca4*)	RrD749F	TGGTAGCATTAAAAGCTGATGG	~ 1,090	Jiang et al. 2005
		RrD1826R	TCTAAATKCTGCTGMATCAAT		
*Coxiella* spp.	16S rRNA	16S rRNA F	GGGGAAGAAAGTCTCAAGGGTAATATCCTT	532	Almeida et al. 2012
		16S rRNA R	TGCATCGAATTAAACCACATGCTCCACCGC		
	*rpoB*	CoxrpoBF2	GGGCGNCAYGGWAAYAAAGGSGT	607–610	Duron et al. 2015
		CoxrpoBR1	CACCRAAHCGTTGACCRCCAAATTG		
		CoxrpoBF3	TCGAAGAYATGCCYTATTTAGAAG	539–542	
		CoxrpoBR3	AGCTTTMCCACCSARGGGTTGCTG		
	*GroEL*	CoxGrF1	TTTGAAAAYATGGGCGCKCAAATGGT	655	Duron et al. 2014
		CoxGrR2	CGRTCRCCAAARCCAGGTGC		
		CoxGrF2	GAAGTGGCTTCGCRTACWTCAGACG	619	
		CoxGrFR1	CCAAARCCAGGTGCTTTYAC		
Anaplasmataceae	16S rRNA	EHR16SD	GGTACCYACAGAAGAAGTCC	345	Parola et al. 2000
		EHR16SDR	TAGCACTCATCGTTTACAGC		
*Anaplasma* spp.	16S rRNA	AnaplsppF	AGAAGAAGTCCCGGCAAACT	~ 800	Zobba et al. 2014
		AnaplR3	GAGACGACTTTTACGGATTAGCTC		
*Babesia* spp.	18S rRNA	F	GTTTCTGMCCCATCAGCTTGAC	422–440	Hilpertshauser et al. 2006
		R	CAAGACAAAAGTCTGCTTGAAAC		
*Borrelia* spp.	16S rRNA	16SF1	ATAACGAAGAGTTTGATCCTGGC	1,350	Masuzawa et al. 1999
		16SR	CAGCCGCACTTTCCAGTACG		
*Hepatozoon* spp.	18S rRNA	HepF300	GTTTCTGACCTATCAGCTTTCGAC G	~ 600	Ujvari et al. 2004
		Hep900	C AAATCTAAGAATTTCACCTCTGAC		
*Wolbachia*	*ftsZ*	ftsF	GTATGCCGATTGCAGAGCTTG	769	Holden et al. 1993
		ftsR	GCCATGAGTATTCACTTGGCT		
Tick	16S rRNA	16S + 1	CTGCTCAATGATTTTTTAAATTGCTGTGG	460	Black and Piesman 1994
		16S-1	CCGGTCTGAACTCAGATCAAGT		

### DNA sequencing and phylogenetic analysis

All positive amplicons were purified using the GF-1 Ambi Clean kit (Vivantis) according to the manufacturer’s instructions and sequenced in both directions on an ABI 3730xl DNA analyzer (Applied Biosystems). All obtained DNA sequences were assembled and edited using BioEdit (Alzohairy 2011). Edited sequences were assembled into a contig using SeqMan software (DNASTAR, Lasergene), and thus were subjected to BLASTn analysis (http://blast.ncbi.nlm.nih.gov/Blast.cgi) to find sequence similarity to known sequences. Phylogenetic analyses were performed using the maximum parsimony method (PAUP v. 4.0b1) and bootstrap analysis was calculated with 1,000 replicates.

## Results

### Identification of ticks

A total of 23 mammals from 8 provinces of Thailand were examined for tick infestation. These mammals belonged to seven species: dogs (*Canis lupus familiaris*), cat (*Felis catus*), sheep (*Ovis aries*), goats (*Capra aegagrus hircus*), cattle (*Bos primigenius taurus*), Asian palm civet (*Paradoxurus hermaphroditus*), and Burmese ferret-badger (*Melogale personata*). In total, 233 tick specimens comprising seven species belonging to three genera were identified as follows: *R. sanguineus* (71/233; 30.5%), *R. microplus* (71/233; 30.5%), *H. bispinosa* (61/233; 26.2%), *H. asiatica* (15/233; 6.4%), *H. heinrichi* (6/233; 2.6%), *H. hystricis* (1/233; 0.4%), and *Dermacentor auratus* (8/233; 3.4%) (Table 2).

**Table 2:** PCR results of *Rickettsia*, *Coxiella*, and *Anaplasma* in mammal ticks collected from 8 provinces in Thailand

Province	Mammal host (No.)	Tick species (No.)	No. of samples tested	PCR results: No. of positive ticks		
				*Rickettsia*	*Coxiella*	*Anaplasma*
1. Chiang Rai	Dog (2)	*Rhipicephalus sanguineus* (22)	12M, 10F	-	-	-
	Cow (2)	*Rhipicephalus microplus* (9)	4M, 5F	-	-	-
	Asian palm civet (1)	*Haemaphysalis asiatica* (15)	9M, 6F	-	6M	-
2. Loei	Burmese ferret-badger (1)	*Haemaphysalis heinrichi* (6)	1M	-	-	1M
			1M	1M*	1M*	-
			2M, 2F	-	-	-
		*Haemaphysalis hystricis* (1)	1M	-	1M	-
		*Dermacentor auratus* (8)	8N	-	-	-
3. Roi Et	Cow (1)	*Haemaphysalis bispinosa* (11)	5M, 6F	-	-	-
	Cow (1)	*Rhipicephalus microplus* (15)	4M, 2F	-	-	-
			1PN (*n*=5)	-	-	-
			1PN (*n*=4)	-	-	-
4. Chumphon	Dog (2)	*Rhipicephalus sanguineus* (18)	9M, 9F	-	-	-
5. Surat Thani	Dog (3)	*Rhipicephalus sanguineus* (21)	7M, 9F	-	-	-
			1PN (*n*=5)	-	-	-
6. Ranong	Dog (2)	*Rhipicephalus sanguineus* (10)	6M, 4F	-	-	-
	Cow (1)	*Rhipicephalus microplus* (15)	4M, 11F	-	-	-
7. Phang Nga	Cow (2)	*Rhipicephalus microplus* (32)	14M, 18F	-	-	-
	Sheep (1)	*Haemaphysalis bispinosa* (11)	2M, 9F	-	-	-
	Goat (1)	*Haemaphysalis bispinosa* (20)	3M, 17F	-	-	-
8. Satun	Cat (1)	*Haemaphysalis bispinosa* (15)	1PN (*n*=3)	-	-	-
			1PN (*n*=3)	-	-	-
			1PL (*n*=5)	-	-	-
			1PL (*n*=4)	-	-	-
	Sheep (1)	*Haemaphysalis bispinosa* (2)	2M	-	-	-
	Goat (1)	*Haemaphysalis bispinosa* (2)	1PN (*n*=2)	-	-	-
Total	23	233	210	1	8	1

*F* female; *M* male; *N* nymph; *PN* pooled nymphs; *PL* pooled larvae; - PCR negative; * co-infection

The results of tick molecular identifications were consistent with morphological identifications using taxonomic key. The partial mitochondrial 16S rRNA gene sequences of ticks were submitted to GenBank under accession numbers as following: ON055731 (*H. asiatica*), ON062951 (*H. hystricis*), and ON074588 (*H. heinrichi*).

### Detection of tick-borne bacteria and protozoa in tick samples

A total of 233 tick specimens were molecularly detected for bacterial and protozoal microorganisms by PCR technique. All tick samples collected from domestic mammals showed PCR negative results while some of the ticks collected from Burmese ferret-badger in Loei province and from Asian palm civet in Chiang Rai province were positive for *Rickettsia* (0.4%; 1/233) and *Coxiella* (3.4%; 8/233) (Table 2). Rickettsial DNA was detected in a male of *H. heinrichi*. *Coxiella*-like endosymbionts were detected in a male of *H. hystricis* (Cox-hys), six males of *H. asiatica* (Cox-asia), and a male of *H. heinrichi* (Cox-hein) which was co-infected with SFG *Rickettsia* sp. Interestingly, *Anaplasma* DNA was detected in another male of *H. heinrichi* infesting Burmese ferret-badger from Loei province (Table 2). None of the 233 mammal ticks gave specific PCR products of *Borrelia*, *Ehrlichia*, *Wolbachia*, *Babesia*, and *Hepatozoon*.

### DNA sequencing and phylogenetic analysis of tick-borne bacteria

The partial sequences of *Rickettsia* sp. in a male of *H. heinrichi* were submitted to GenBank under accession numbers MW415893 (17-kDa antigen), MW415895 (*gltA*), MW415897 (*ompA*), OK031073 (*ompB*), and OK031072 (*sca4*). The DNA sequences of CLE detected in *H. heinrichi* (Cox-hein), *H. hystricis* (Cox-hys), and *H. asiatica* (Cox-asia) were submitted to GenBank under accession numbers: MW404679, MW404677, and MW404676 for partial 16S rRNA; OK031069, OK031070, and OK031071 for partial *GroEL*; OK031066, OK031067, and OK031068 for partial *rpoB*. The GenBank accession number of partial 16S rRNA of *Anaplasma* sp. detected from a male of *H. heinrichi* was MW405449.

**Fig. 1:**
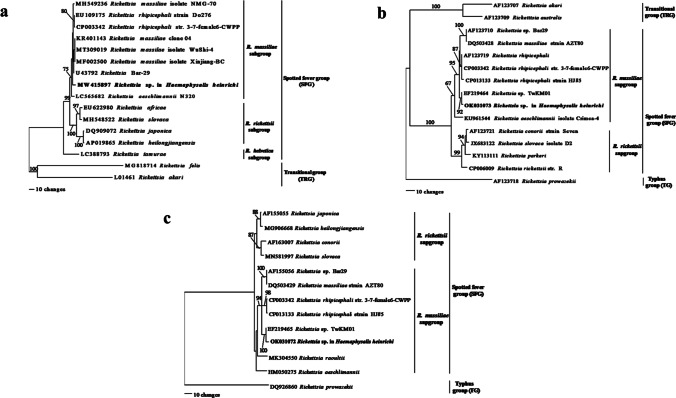
Maximum parsimony tree of *Rickettsia* spp. based on the partial sequences of *ompA* (**a**), *ompB* (**b**), and *sca4* (**c**) genes. The bootstrap values are shown above or near branch. *Rickettsia* spp. detected in present study are indicated in bold

The BLAST result of partial sequences of 17-kDa amplified from a male of *H. heinrichi* indicated a high nucleotide sequence similarity (99.08–99.54%) to SFG rickettsiae members, namely *R. rhipicephali* (MN477896), *R. massiliae* (KY069262), and *R. raoultii* (MW321554). Likewise, the BLAST result of partial *gltA* derived from the same tick showed high similarity (98.17–98.69%) to *R. rhipicephali* (KX018048), *R. massiliae* (KY640405), and other members of SFG rickettsiae, including *R. raoultii*, *R. japonica*, and *R. heilongjiangensis*. However, a more variable gene, *ompA* partial sequence obtained from this tick, was highly similar (98.12–98.31%) only to members of *R. massiliae* subgroup: *R. rhipicephali* (CP003342) and *R. massiliae* (MT309019, MW779485, etc.). Correspondingly, the results for partial *ompB* and *sca4* sequences demonstrated the highest similarity to members of *R. massiliae* subgroup; 96.65% with *R. rhipicephali* (AF123719, CP003342) and 96.97% with *R. massiliae* (CP003319, DQ503429). Phylogenetic trees inferred from partial sequences of these genes revealed three groups of rickettsiae, comprising SFG, TRG, and TG. The SFG rickettsiae sequences utilized in phylogenetic analysis contained two subgroups: *R. rickettsii* and *R. massiliae*. *Rickettsia* sp. detected in this study was grouped in the SFG clade, clustered with members of the *R. massiliae* subgroup, including *R. rhipicephali*, *R. massiliae*, *R. aeschimannii*, *Rickettsia* sp. TwKM01, and *Rickettsia* sp. Bar29 (Fig. 1).

**Fig. 2:**
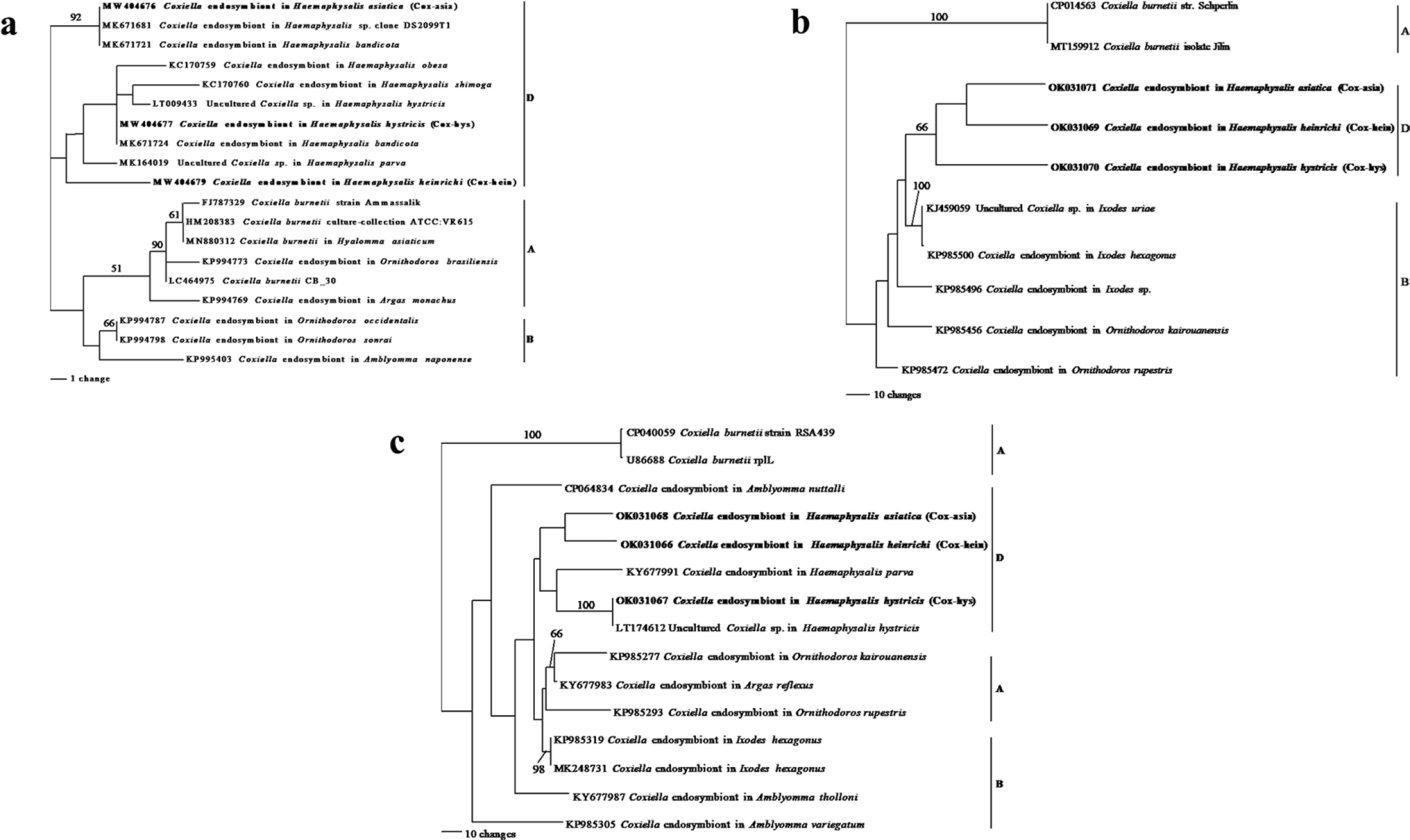
Maximum parsimony tree of *Coxiella* spp. based on the partial sequences of 16S rRNA (**a**), *GroEL* (**b**), and *rpoB* (**c**) genes. The bootstrap values were shown above or near the branch. *Coxiella* endosymbionts detected in this study are indicated in bold. Three clades of *Coxiella* are indicated as A, B, and D which were originally defined by Duron et al. 2015

Based on three partial sequences of 16S rRNA, *GroEL*, and *rpoB*, all *Coxiella* spp. amplified from three *Haemaphysalis* tick species were CLE. In contrast, *C. burnetii* was not detected in this study. Nucleotide sequences of the three CLE (Cox-hein, Cox-hys, and Cox-asia) were different from each other. The BLAST results of these *Coxiella* partial 16S rRNA sequences indicated that their nucleotide sequences were highly similar to other CLE of several *Haemaphysalis* tick species represented in GenBank (98.52–100%). However, BLAST results of partial *GroEL* and *rpoB* sequences showed lower level of nucleotide sequence similarity to other CLE available in GenBank than that found with 16S rRNA. The nucleotide sequence similarity of CLE detected in this study was 89.42–93.64% for *GroEL* and 91.04–100% for *rpoB*. Phylogenetic analysis of partial 16S rRNA revealed that Cox-hein and Cox-hys in ticks removed from Burmese ferret-badger were grouped in the same subclade of clade D, while Cox-asia in ticks removed from Asian palm civet was placed in another subclade (Fig. 2). In contrast, the phylogenetic trees generated from the partial sequences of *GroEL* and *rpoB*, Cox-hein were more closely related to Cox-asia than to Cox-hys (Fig. 2). However, all three CLE detected in this study were grouped in clade D with other CLE of *Haemaphysalis* ticks from previous reports.

**Fig. 3:**
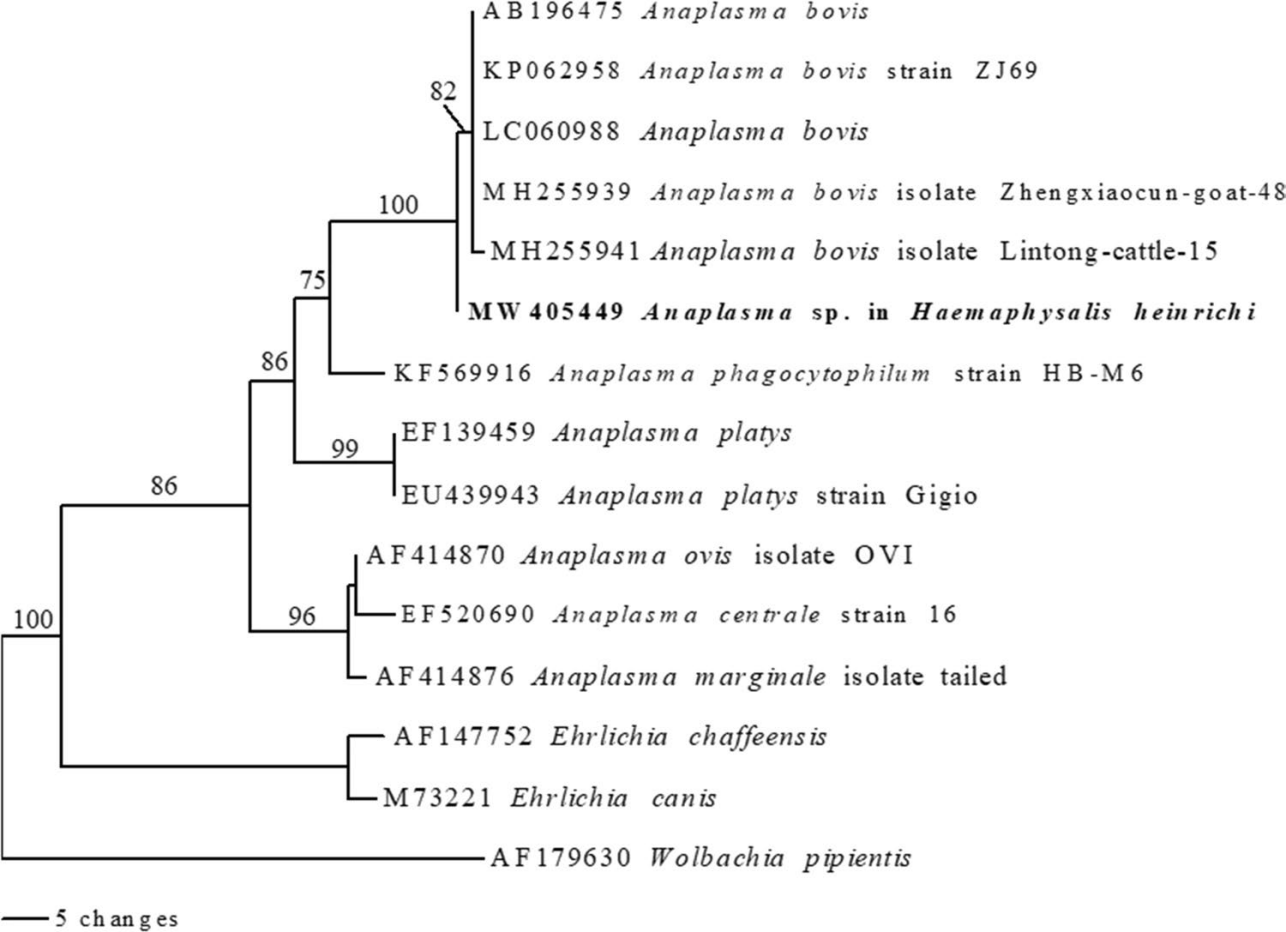
Maximum parsimony tree of *Anaplasma* spp. based on the partial 16S rRNA gene sequences. The bootstrap values are shown above the branch. The *Anaplasma* sp. sequence obtained in present study is indicated in bold

*Anaplasma* sp. was found only in a male of *H. heinrichi* using PCR with 16S rRNA primers. BLAST results of the partial sequences of 16S rRNA illustrated a remarkably high similarity (99.73%; 730/732 bp) to several strains of *A*. *bovis*, for example, Zhengxiaocon-goat-48 (MH255939), ZJ69 (KP062958), sika35 (LC060988), NR07 (AB196475), and many other strains. Based on phylogenetic analysis of partial 16S rRNA gene sequences, *Anaplasma* sp. detected in this study manifestly belonged to the same clade with *A*. *bovis* with strong bootstrap support (100%) (Fig. 3). The results of BLAST and phylogenetic analysis thus explicitly demonstrated that a male of *H. heinrichi* was infected with *Anaplasma* sp. closely related to *A. bovis*.

## Discussion

Ticks serve as vectors and/or reservoirs of several pathogens such as *Anaplasma*, *Coxiella*, *Ehrlichia*, and *Rickettsia* (Walker and Yu 2012). To better understand tick-associated microorganisms among various mammal ticks in Thailand, we conducted specific molecular screening for the presence of bacterial and protozoal microorganisms. Previous studies showed that *Rickettsia* spp. were widely distributed throughout Thailand in various locations, hosts, and in tick species, including *H. ornithophila*, *Amblyomma testudinarium*, *H. shimoga*, *H. lagrangei*, *A. helvolum*, and *A. varanense* (Hirunkanokpun et al. 2003; Ahantarig et al. 2011; Sumrandee et al. 2014; Malaisri et al. 2015; Nooroong et al. 2018). In this study, a SFG *Rickettsia* sp. was detected in a male of *H. heinrichi* infesting Burmese ferret-badger in Loei province, northeastern Thailand. The members of SFG rickettsiae have been categorized into four subgroups: *R. rickettsii*, *R. massiliae*, *R. helvetica*, and *R. akari* (Merhej and Raoult 2011). Our results revealed five partial rickettsial sequences of 17-kDa, *gltA*, *ompA*, *ompB*, and *sca4* genes that were amplified and sequenced from a *H. heinrichi* tick. The analysis of partial 17-kDa and *gltA* sequences indicated that the *Rickettsia* sp. was a member of SFG rickettsiae. However, these two genes could not differentiate whether it belonged to the *R. massiliae* or *R. rickettsii* subgroups. Since 17-kDa and *gltA* genes are highly conserved among SFG rickettsiae, previous studies have suggested that less conservative rickettsial genes such as *ompA*, *ompB*, and *sca4* should be more suitable for the comparisons of closely related SFG species (Robinson et al. 2019). Therefore, these gene sequences were used for characterization of *Rickettsia* sp. in this study. BLAST and phylogenetic analyses of the partial *ompA*, *ompB*, and *sca4* genes showed that the detected SFG *Rickettsia* sp. found in this study was a member of *R. massiliae* subgroup and was closely related to both *R. rhipicephali* and *R. massiliae.* However, it remains uncertain whether this *Rickettsia* sp. is pathogenic *R. massiliae* or non-pathogenic *R. rhipicephali.* Further characterizations by amplification and sequencing of additional genes, such as *sca2* and *GroEL*, are required to clarify this point. In Thailand, a *Rickettsia* sp. closely related to members of the *R. massiliae* subgroup has been reported in *H. lagrangei* collected from sambar deer (Sumrandee et al. 2016). We reported herein for the first time SFG *Rickettsia* sp. belonging to the *R. massiliae* subgroup in *H. heinrichi* infesting a wild Burmese ferret-badger.

The distribution and diversity of tick species may be crucial to the investigation of tick-borne diseases. Cornet et al. (2009) indicated that *Haemaphysalis* was one of the most common genera widely distributed throughout Thailand and was capable of transmitting tick-borne diseases to humans. Humans bitten by infected *Haemaphysalis* ticks are consequently exposed to a high risk of bacterial infection. *Haemaphysalis heinrichi* was previously found infesting four species of mammal hosts: *Arctonyx collaris*, *Bos domesticus*, *Canis familiaris*, and *Melogale personata* in Chiang Mai, Chiang Rai, Khon Kaen, Bangkok, Nakhon Ratchasima, Prachinburi, and Ubon Ratchathani (Tanskul et al. 1983). Our findings have provided additional evidence of *H. heinrichi* infesting Burmese ferret-badger in Loei province, northeastern Thailand. Further study of the abundance and distribution of *H. heinrichi* and related tick species and the prevalence of bacterial infection will provide a better understanding of the epidemiology of rickettsioses and other tick-borne diseases in Thailand.

*Coxiella*-like endosymbionts have frequently been found in ticks (Ahantarig et al. 2011; Almeida et al. 2012; Arthan et al. 2015; Duron et al. 2015). Multilocus sequence analysis of five *Coxiella* housekeeping genes indicated that *Coxiella* endosymbiont in ticks served as the common ancestor of *C. burnetii* (Duron et al. 2015). The endosymbiont may play a vital role in providing vitamin and cofactor biosynthesis pathways and in defining the reproductive fitness of tick hosts (Smith et al. 2015). Consequently, understanding its role in sustaining growth and survival of ticks may provide new methods for control and management of tick populations. Q fever and seroprevalence of *C. burnetii* have been reported in rural areas of Malaysia and Thailand (Suputtamongkol et al. 2003; Bina Rai et al. 2011). In Malaysia, both *C. burnetii* and *Coxiella* endosymbiont were reported in *H. hystricis* ticks collected from the same wild boar. In this study, *C. burnetii* was not detected in all tick samples and CLE were detected solely in one (*Haemaphysalis*) of three analyzed tick genera. We identified three different types of CLE, namely Cox-hein in *H. heinrichi*, Cox-hys in *H. hystricis*, and Cox-asia in *H. asiatica*. Phylogenetic analysis with five concatenated gene sequences by Duron et al. (2015) showed that the genus *Coxiella* was divided into four clades (A-to-D) corresponding to the host genera. In this study, the phylogenetic trees generated from partial 16S rRNA, *GroEL*, and *rpoB* gene sequences demonstrated that the three CLE were clustered with those of *Haemaphysalis* ticks and were placed in clade D. The result of CLE partial 16S rRNA sequences obtained in this study was in accordance with previous authors who suggested that CLE detected from the same host genera were clustered within the same clade (Khoo et al. 2016; Trinachartvanit et al. 2018). However, *GroEL* and *rpoB* gene sequences did not cluster together with those previously reported in the same host genera (Khoo et al. 2016; Trinachartvanit et al. 2018). In Thailand, co-infections of *Rickettsia* and *Coxiella* have been reported in *H. lagrangei* and *A. testudinarium* (Nooroong et al. 2018; Sumrandee et al. 2016). Our results have also demonstrated the first case of co-infection of CLE and SFG *Rickettsia* sp. closely related to *R. massiliae* subgroup in *H. heinrichi* infesting Burmese ferret-badger. Interestingly, many wild animals have been reported as reservoirs of pathogenic bacterial agents of humans, including *A. phagocytophilum*, *C. burnetii*, and *Rickettsia* spp. (Meerburg and Reusken 2011; Silaghi et al. 2014). Further investigation in this field of research will provide more information on the prevalence of tick-borne pathogens in various tick species infesting wild mammals in Thailand before any conclusion can be made.

Anaplasmosis is an infectious hemotropic disease of cattle, sheep, goats, and other ruminants worldwide. Several *Anaplasma* species are causative agents of anaplasmosis in ruminants, e.g., *A. phagocytophilum*, *A. marginale*, and *A. bovis* (Inokuma 2007). In Thailand, *Anaplasma* spp. have been reported in both domestic and wild mammals and their associated ticks, including *A. platys* which have been detected from blood of mammal hosts i.e., cats, dogs, rodents, sambar deer, and wild boar, or from their parasitic ticks (Parola et al. 2003; Pinyoowong et al. 2008; Foongladda et al. 2011; Salakij et al. 2012; Sumrandee et al. 2016). *Anaplasma marginale* and *A. phagocytophilum* were found in water buffalo and ticks collected from vegetation, respectively (Nguyen et al. 2020; Nooroong et al. 2018); *A. bovis* has been reported in ticks infesting bear, sambar deer, rodents, and ticks collected from vegetation (Parola et al. 2003; Malaisri et al. 2015; Sumrandee et al. 2016; Takhampunya et al. 2019). In this study, an *Anaplasma* sp. closely related to *A. bovis* was detected in *H. heinrichi* removed from Burmese ferret-badger. Thus, the results of the present and previous studies in Thailand demonstrated that *A. bovis* were commonly found in ticks parasitizing domestic and wild mammals.

Ixodid ticks of the genera *Ixodes, Dermacentor*, *Rhipicephalus*, and *Amblyomma* are the main vectors of *Anaplasma* bacteria (Dumler et al. 2001). However, this bacterium has also been found in *Haemaphysalis* ticks in Asia and North America (Goethert and Telford 2003; Kim et al. 2003; Qin et al. 2018; Fukui and Inokuma 2019). In Thailand, *Anaplasma* spp. have been detected in ticks such as *A. platys* in *D. auratus* (Parola et al. 2003) and *R. sanguineus* (Foongladda et al. 2011); *A. phagocytophilum* in *D. auratus* (Nooroong et al. 2018); *A. bovis* in *H. lagrangei* (Parola et al. 2003; Sumrandee et al. 2016), *H. shimoga* (Malaisri et al. 2015), *H. obesa* (Sumrandee et al. 2016), and *H. bandicota* (Takhampunya et al. 2019). In this study, the presence of *Anaplasma* sp. closely related to *A. bovis* in *H. heinrichi* has been demonstrated by sequence and phylogenetic analysis of partial *Anaplasma* 16S rRNA. Our findings support previous studies in Thailand and other countries in Asia in which *A. bovis* is mostly found in *Haemaphysalis*, and this tick genus also associates with the transmission of *Anaplasma* (Goethert and Telford 2003; Parola et al. 2003; Lee and Chae 2010; Yoshimoto et al. 2010; Malaisri et al. 2015; Sumrandee et al. 2016). To our knowledge, this is the first report of *Anaplasma* sp. closely related to *A. bovis* in *H. heinrichi* tick-infested Burmese ferret-badger.

In summary, we identified tick-borne bacteria among various mammal ticks collected from 8 provinces of Thailand. Based on DNA sequencing and phylogenetic analyses, we found SFG *Rickettsia* sp. in the *R. massiliae* subgroup in *H. heinrichi* and three CLE, namely Cox-hein, Cox-hys in *H. heinrichi* and *H. hystricis* ticks infesting a Burmese ferret-badger, and Cox-asia in *H. asiatica*. Co-infection of SFG *Rickettsia* sp. and CLE (Cox-hein) was detected in *H. heinrichi*. This study also provided the first evidence for the presence of an *Anaplasma* sp. closely related to *A. bovis* in a *H. heinrichi* tick. Our results extended the knowledge of geographic distribution of ticks parasitizing different species of mammals and vector-borne bacteria in Southeast Asia.

## Data Availability

All data included in this study are available on request to the corresponding author.
