# Correction to: Spotted fever group *Rickettsia*, *Anaplasma* and *Coxiella*‑like endosymbiont in *Haemaphysalis* ticks from mammals in Thailand

**DOI:** 10.1007/s11259-022-09988-3

**Published:** 2022-08-22

**Authors:** Supanee Hirunkanokpun, Arunee Ahantarig, Visut Baimai, Pairot Pramual, Pakavadee Rakthong, Wachareeporn Trinachartvanit

**Affiliations:** 1 Department of Biology, Faculty of Science, Ramkhamhaeng University, Bangkok, 10240 Thailand; 2 Biodiversity Research Cluster, Department of Biology, Faculty of Science, Mahidol University, Bangkok, 10400 Thailand; 3 Department of Biology, Faculty of Science, Mahasarakham University, Maha Sarakham, 44150 Thailand; 4 Faculty of Science and Technology, Rajabhat Suratthani University, Surat Thani, 84100 Thailand

**Correction to: Veterinary research communications**

**https://doi.org/10.1007/s11259-022-09980-x**

In the published paper, the 4th author should be Pairot Pramual.

The original article has been corrected.

